# Subjective cognitive complaints and relations to objective cognitive performance among Lebanese patients with schizophrenia

**DOI:** 10.1186/s12888-021-03564-w

**Published:** 2021-11-09

**Authors:** Chadia Haddad, Pascale Salameh, Hala Sacre, Clément Polin, Jean-Pierre Clément, Benjamin Calvet

**Affiliations:** 1 Research department, Psychiatric Hospital of the Cross, Jal Eddib, Lebanon; 2 INSPECT-LB (Institut National de Santé Publique, d’Épidémiologie Clinique et de Toxicologie-Liban), Beirut, Lebanon; 3 INSERM, Univ. Limoges, IRD, U1094 Tropical Neuroepidemiology, Institute of Epidemiology and Tropical Neurology, GEIST, 87000 Limoges, France; 4 Pôle Universitaire de Psychiatrie de l’Adulte, de l’Agée et d’Addictologie, centre hospitalier Esquirol, 87025 Limoges, France; 5 Faculty of Pharmacy, Lebanese University, Beirut, Lebanon; 6 University of Nicosia Medical School, Nicosia, Cyprus; 7 Centre mémoire de ressources et de recherche du Limousin, centre hospitalier Esquirol, 87025 Limoges, France; 8 Unité Recherche et Innovations, centre hospitalier Esquirol, 87025 Limoges, France

**Keywords:** Insight, Schizophrenia, Subjective, Cognition, Complaint

## Abstract

**Background:**

Patients with schizophrenia have a particularly low level of insight into their illness compared to people with other mental health disorders. The objectives of the study were to evaluate: 1) subjective cognitive complaints in individuals with schizophrenia in comparison with health controls, 2) the relation between subjective cognitive complaint (SCC) and objective cognitive performance in the patients group, and 3) factors related to cognitive complaint, such as depression, insight, autonomy, and psychological symptoms.

**Methods:**

Cross-sectional study was conducted between July 2019 and March 2020 enrolled 120 patients with schizophrenia disorders, selected from the Psychiatric Hospital of the Cross (HPC) – Lebanon and 60 healthy controls. The Self-Assessment Scale of Cognitive Complaints in Schizophrenia (SASCCS) was used to measure people living with schizophrenia perception of their cognitive impairment, while the Brief Assessment of Cognition in Schizophrenia (BACS) was used to evaluate their cognitive functioning.

**Results:**

A significant difference was found between schizophrenia patients and healthy controls in all neurocognition and SASCCS tests. The hierarchical regression analysis showed that the BACS total score (Beta = −.06, *p* = .04), the PANSS general psychopathology (Beta = .29, *p* = .003), higher depression (Beta = .75, *p* = .003) were significantly associated with higher SCC. However, higher autonomy (Beta = − 6.35, *p* = .001) was significantly associated with lower SCC. A Structural equation model showed that the two most contributing variables were general psychopathology (Standardized Beta (SB): .33, *p* < 0.001) and autonomy (SB: −.29, *p <* 0.001).

**Conclusion:**

A significant proportion of patients with schizophrenia could estimate their cognitive impairment. It also showed a positive correlation between depression and activity of daily living with SCC, suggesting that this aspect should be investigated alongside the clinical symptoms when a patient with schizophrenia presents with SCC.

**Supplementary Information:**

The online version contains supplementary material available at 10.1186/s12888-021-03564-w.

## Background

Subjective cognitive complaints (SCC) are the self-perception of cognitive functioning, such as difficulties with concentration, memory, decision making, and clear thinking. Patients with schizophrenia have a particularly low level of insight into their illness compared to people with other mental health disorders [1]. This aspect of insight, termed neurocognitive insight, is described as the “awareness of neuropsychological dysfunction” [2]. If patients could reliably report their own cognitive issues, this could help with the planning of functional outcomes and individual treatments. Some studies could not find an association between SCC and objective cognition assessed with neuropsychological tests [3, 4], while others found a weak association [5–7]. The latter studies support the notion that schizophrenia patients are known to show only a partial understanding of their cognitive deficits [8]. Therefore, clinicians rely on neuropsychological assessments to determine the cognitive status of the patients rather than self-report measures [9]. Additionally, SCC were more frequently reported in patients with schizophrenia compared to healthy controls [10]. Few studies have shown that neither in patients with schizophrenia nor in stable controls, subjective cognitive impairment scores were associated with objective cognition [2, 11]. Hamilton had demonstrated that self-rated memory questionnaires provided to healthy subjects have no relation to actual memory capability in the general population [12]. Thus, it seems that lack of insight into cognitive deficits is not just a specific aspect in patients with schizophrenia.

Several factors showed to be associated with SCC, such as insight, psychotic symptoms, depression, and medication side effects. Indeed, some studies revealed significant relationships between psychiatric symptoms of schizophrenia and cognitive complaints of patients, which suggests that cognitive complaints might reflect, in schizophrenia, an expression of diffuse illness rather than real awareness of their cognitive deficits [4, 13, 14]. However, other studies found contradictory results, showing no relationship between SCC and the severity of psychopathology [8, 15]. Also, depression showed to be correlated with an underestimation of one’s mental ability and thus with more subjectively perceived difficulties in cognitive processing problems [15]. Patients with depressive symptoms have difficulty concentrating while working and remembering things. In a study done by Harvey et al. among 406 people living with schizophrenia have showed that participants with very low self-reported depression overestimated their everyday functioning [16]. In addition, the existence of autistic characteristics is another possible correlate of reduced self-assessment abilities. People with these characteristics might have a lot of social detachment and have trouble judging whether or not their behaviors are socially suitable. In a study done by Harvey et al. among 177 patients living with schizophrenia have found that autistic traits were associated with impairments (underestimation) in self-assessment of everyday functioning [17]. Moreover, lack of insight in patients with schizophrenia is linked to poor estimation of their cognitive deficits that affect all stages of the clinical process, from referral to monitoring progress and assessing improvement. Medication side effects are also associated with cognition; studies have found that higher doses of conventional antipsychotics were related to decreased subjective cognitive well-being [18]. Also, patients tolerate better atypical antipsychotics, where higher doses of these medications were not related to neurocognitive functioning [18].

Several subjective scales have been used to measure cognitive complaints in people living with schizophrenia, including the Bonn Scale [19], the Frankfurt Complaint Scale [20], the Subjective Experience of Deficits in Schizophrenia (SEDS) [21], the subjective experience interview [22], the Subjective Deficit Syndrome Scale [23], and the Eppendorf Schizophrenia Inventory (ESI) [24]. These tools addressed different schizophrenia subjective experiences, including cognitive dysfunctions, but did not focus on this particular aspect. Hence, two scales were developed to evaluate cognitive dysfunctions, i.e., the Subjective Scale To Investigate Cognition in Schizophrenia (SSTICS) [7] and the Self-Assessment Scale of Cognitive Complaints in Schizophrenia (SASCCS) [13]. The latter, created in the Tunisian Arabic dialectic language, consists of 21 questions addressing the five cognitive domains most frequently reported in the literature to be impaired in schizophrenia: memory, attention, executive functions, language, and praxis. It was designed to be clear, simple, and easy to use by patients with schizophrenia [13].

Cognitive self-assessment impairments were shown to be more strongly linked to poor everyday functioning than performance on neurocognitive testing. Although the number of publications on cognitive deficits in schizophrenia has substantially increased over the past two decades, little is known about how patients with schizophrenia perceive their own cognition. To the best of our knowledge, no studies in the Arab countries or Lebanon have been conducted to assess subjective cognitive complaint in institutionalized individuals with neurological or psychiatric diseases. In addition, clinicians in hospitals and clinics often underestimate the degree of cognitive impairment in people living with schizophrenia and misestimate the ability of patients to evaluate their cognitive deficits. Also, few studies have investigated the predictive values of insight, depression, and symptoms of SCC, or whether they contribute individually or not to the estimation of SCC. Therefore, it is essential to study the SCC among institutionalized schizophrenia patients as accelerated stage of cognitive decline have been demonstrated among this group [25]. It is also important to know if the clinical symptoms, insight, autonomy and depression would predict SCC. Self-assessment of cognition enables one to become aware of their symptoms and address the negative effects in everyday life. It improves the therapeutic connection and the patient’s desire to receive care, especially in cognitive rehabilitation [26]. Understanding the relationship between subjective and objective measures of cognitive dysfunction and factors related to SCC in schizophrenia would help the treatment guidance in clinical settings.

Therefore, the study objectives were to evaluate: 1) subjective cognitive complaints in individuals with schizophrenia in comparison with health controls, 2) the relation between subjective cognitive complaint and objective cognitive performance in the patients group, and 3) factors related to cognitive complaint, such as depression, insight, autonomy, and psychological symptoms.

## Methods

### Study design and participants

A cross-sectional study conducted between July 2019 and March 2020 enrolled 120 chronic in-patients with schizophrenia disorders, selected from the Psychiatric Hospital of the Cross (HPC) – Lebanon, and 60 healthy controls. The latter group consisted of the HPC staff and matched with schizophrenia patients in terms of age, education level, and gender. Inclusion criteria were: age between 18 and 60 years, having completed at least 5 years of education, meeting the DSM-5 criteria for schizophrenia conditions, taking an antipsychotic drug, and being clinically stable. The clinical stability of the patients was defined as: “the period during which psychotic symptoms are less severe and the patient is on adequate treatment for at least last 6 months and did not require any increase in dose of antipsychotic medication over last 3 months” [27]. The lack of a diagnosis of significant mental disorders was considered an eligibility factor for healthy controls. Brain trauma, neurological disorders, and existing substance use disorders that would affect cognitive function were exclusion factors for all participants. Those who agreed to take part in the study were asked to sign a written informed consent form and received no compensation in any way. Well-trained, study-independent staff conducted personal interviews for data collection.

### Ethical approval

The study protocol was approved by the Ethics and Research Committee at the Psychiatric Hospital of the Cross, in accordance with the Hospital’s Regulatory Research Protocol (HPC-024-2018). Consent was obtained as written approval on the ethical informed consent form. The procedures used in this study adhere to the tenets of the Declaration of Helsinki.

### Measures

The questionnaire used was in Arabic, the native language in Lebanon. The first section analyzed the sociodemographic and clinical characteristics of participants, including age, gender, education level, marital status, monthly salary, family history of mental illnesses, types of schizophrenia, hospitalization length, illness duration, and the number of hospitalizations. The medications that patients were taking were retrieved from their medical records. The types of medication used by the participants were atypical antipsychotics, typical antipsychotics, mood stabilizers, benzodiazepines, antiepileptics, anticholinergics, and antidepressants (Selective Serotonin Reuptake Inhibitors-SSRIs and Tricyclic antidepressants-TCAs), in addition to other medications: anticoagulants, antimuscarinics, antiarrhythmics, antiparkinsonians, antiasthmatics, thyroid medication, stomach protection, antidiabetics, statins, antihypertensives, proton-pump inhibitors, and vitamins and supplements. For antipsychotics, the chlorpromazine dose equivalent was calculated using the Andreasen method [28].

The second section of the questionnaire included the following measures:

#### The self-assessment scale of cognitive complaints in schizophrenia (SASCCS)

The SASCCS is a self-reported questionnaire used to measure people living with schizophrenia perception of their cognitive impairment [29]. The scale was constructed and validated among 105 people living with schizophrenia recruited from three different outpatient clinics in Tunis [29]. The results showed that a good internal consistency (Cronbach alpha = .85) and the intra-class correlation coefficient was equal to 0.77 [29]. The Principal component analysis revealed a six factors accounting for 58.28% of the total variance of the scale [29]. The scale consists of 21 items rated on a 5-point Likert scale, covering memory (6 questions: 1–3 and 9–11), attention (5 questions: 12–16), executive functions (3 questions: 17–19), language (2 questions: 20–21), and praxia (5 questions: 4–8) [29]. Praxia refer to a motor activity. It is the ability to conceptualize, plan, and organize movements in order to complete unfamiliar motor tasks. Example given of the praxia items: “Do you have any problems to remember the name of your treatments?”; “Have you ever forgotten an appointment with your friend or with your doctor?” and “Have you ever forgotten how to cook a dish or which ingredients you should put in / Have you ever forgotten how to fix or repair things at home”. According to the original article [29], the SASCCS was divided in 6 subjective domains: distractibility, daily life, semantic memory, disorder consciousness, working memory and executive skills. The daily life subscale referred to the ability of the patient to perceive any difficulty remembering or memorizing activities in everyday life. The disorder consciousness subscale refer to the ability of patients to be conscious about their cognitive deficit. The SASCCS items are rated on a 5-point Likert scale from 0 (never) to 4 (very often). The SASCCS total score is calculated by summing all answers, with higher scores indicating higher cognitive impairment complaints [29]. The Cronbach’s alpha value was .911.

#### The brief assessment of cognition in schizophrenia (BACS)

The BACS, recently validated in Lebanon [30], is a neuropsychological battery used to evaluate cognitive functioning in patients with schizophrenia [31]. The Arabic BACS scale was recently validated among 120 people living with schizophrenia where a high Cronbach’s alpha was found (α = .853) and the factor analysis showed a one-factor solution with a variance of 64.8% [31]. A good concurrent validity was found between the BACS composite score and a standard battery composite scores in people living with schizophrenia (*r* = .73, *p* < .001) [31]. The BACS consists of six subscales, i.e., list learning (verbal memory), digit sequencing (working memory), token motor task (psychomotor function), semantic fluency (verbal fluency), symbol coding (attention and speed of information processing), and Tower of London (executive function) [31]. The Cronbach’s alpha value was .853.

#### The positive and negative syndrome scale (PANSS)

The PANSS, validated in Arabic [32], is a 30-item questionnaire organized into three subscales: positive symptoms (7 items), negative symptoms (7 items), and general psychopathology (16 items) [33]. All items are scored from 1 (absence of symptoms) to 7 (extremely severe symptoms) [33]. The total score was calculated by summing all answers, with higher scores indicating more severe symptoms [33]. The PANSS scale was validated in Lebanon among 400 participants (200 people living with schizophrenia and 200 healthy controls) [32]. The results showed that the PANSS scale items converged over a solution of three factors, explaining a total of 64.81% of the variance [32]. A high Cronbach’s alpha was found for the full scale (.961), the positive symptoms (.877), negative symptoms (.933) and general psychopathology (.926) [32]. In this study, the Cronbach’s alpha values were as follows: .684 (total score), .769 (positive symptoms), .778 (negative symptoms), and .836 (general psychopathology).

#### Calgary depression scale for schizophrenia (CDSS)

The CDSS is a 9-item structured interview scale developed by Addington et al. [34] to assess depression in patients with schizophrenia. The Arabic version of the CDSS was validated among 204 subjects recruited from the Arab population residing in Doha, Qatar (102 people living with schizophrenia and 102 controls subjects) [35]. The CDSS showed good internal consistency (Cronbach’s alpha = .82) [35]. The Intraclass Coefficient correlations (ICC) for the inter-rater reliability was .90, *p* < .05 and test-retest reliability was .85, *p* < .001 [35]. A high sensitivity 72.75% and specificity 67.95% was found [35]. Eight structured questions assess depression, hopelessness, self-depreciation, guilty ideas of reference, pathological guilt, morning depression, early wakening, and suicide, followed by one observation item (observed depression). Higher scores represent a greater level of depression [34]. The Cronbach’s alpha value was .839.

#### Activities of daily living (ADL)

The ADL is a 6-item scale that assesses overall functional activity in: 1) bathing, 2) dressing, 3) going to the toilet, 4) transferring (movement), 5) continence, and 6) feeding [36]. Nasser and Doumit had validated the Arabic version of the scale in a 354 Lebanese elderly living in nursing homes [37]. The results of the latter study showed that the reliability split half measures, sensitivity, and negative predictive values were high across all dimensions of the ADL with the exception of feeding [37]. The reliability split half measure had a strong Cronbach alpha of 0.90 for the first three subscales on the ADL (bathing, dressing, and going to the toilet) [37]. The Cronbach alpha for the second three subscales was 0.65 for transferring (movement), continence, and feeding, with a correlation of *r* = .8 between the two halves [37]. In the Arabic version, the six components are scored 0, .5, or 1. The total ADL score ranges from 0 to 6, where 6 entails complete independence and 0 complete dependence. The total mean score was calculated by summing the scores of the six items, where a higher score indicates a greater autonomy level. The Cronbach’s alpha value was .684.

#### Insight scale for psychosis (IS)

This 8-item self-report questionnaire measures insight in patients with psychotic disorders [38]. The validation of the scale was done among 30 patients recruited during an admission for acute psychotic relapse [38]. The Cronbach alpha of the total scale was .75, the test – retest reliability was .90 [38]. The results of the factor analysis showed that one factor was extracted that accounted for 60% of the variance [38]. The eight items are organized into three subscales (awareness of illness, re-labeling of symptoms, and need of treatment), each with a mean score from 0 to 4. The total score calculated by summing subscale scores ranges from 0 to 12. The higher the score, the greater the insight. The Cronbach’s alpha value for the total scale was .503.

### Translation procedure

The SASCCS and IS scales were translated from English into Arabic using the forward and backward translation method. Each procedure was performed by a different translator. Discrepancies were resolved by consensus between the original English version and the translated one.

### Data analysis

The SPSS software version 25 was used to perform the data analysis. The Shapiro Wilk test was used to check the normality distribution of the SASCCS scale and showed that the main dependent variable was normally distributed. A descriptive analysis was performed, where categorical variables were expressed as absolute frequencies and percentages and quantitative variables as means and standard deviations. The independent-sample t-test was used to compare continuous variables between groups, whereas the ANOVA was used to compare three or more means. Pearson correlation test was used to evaluate the association between continuous variables.

BACS and SASCCS composite scores (z-score) were calculated by averaging the total score from the mean total score of these scales of a healthy control group.

Construct validity of the scales used was assessed using a principal component analysis. To ensure the model’s adequacy, Kaiser-Meyer-Olkin measure of sampling adequacy and Bartlett’s test of sphericity were calculated. Factors with eigenvalues values larger than one were retained and the scree plot method was used for determining the number of components to extract [39]. Only items with factor loading larger than .4 were considered [40]. Moreover, the internal consistency of the scales was assessed using Cronbach’s alpha. The results of the factor analysis was presented in the supplementary file, Tables 1, 2, 3 and 4.

Among people living with schizophrenia, a three-stage hierarchical linear regression analysis was performed to examine the additional variance of each factor on the SASCCS scale. The BACS total score was entered in the first model, clinical symptoms and insight in the second model and depression and autonomy in the last model. Age, gender, and education were entered as covariates into the models.

A structural equational model (SEM) was constructed (using AMOS version 24) to evaluate the relationships between objective cognition, clinical symptoms, depression, insight, autonomy, and subjective cognitive complaint. The following goodness-of-fit indicators were reported: the chi square to df ratio (χ2/df), the Root Mean Square Error of Approximation (RMSEA), the Goodness-of-fit statistic (GFI), the adjusted goodness-of-fit statistic (AGFI) and the comparative fit index (CFI). Significance was set at a *p* < 0.05.

This study is a part of a large project and the same method was used from previous study [30].

## Results

### Sample characteristics

Table 1 shows the sociodemographic characteristics of patients with schizophrenia and healthy controls. Within the Schizophrenia group, more than half of the participants were male (59.2%), single (81.9%), with a secondary level of education (50.0%), and 36.5% had a family history of psychiatric illness. Mean illness and hospitalization lengths were 20.6 ± 12.4 and 12.4 ± 8.5 years, respectively. The mean number of hospitalizations was 6.3 ± 5.65 times, and the mean age was 48.4 years. Within the healthy controls group, the majority were male (60%), married (86.7%), with a low income (67.2%), and 45.0% had a secondary level of education. Only 8.5% had a family history of psychiatric illness. When comparing both groups, schizophrenia patients were more likely to be single, with no income, and a history of psychiatric illness.

**Table 1:** Sociodemographic and clinical characteristics of the total sample (*N* = 180)

	People living with schizophrenia (***N*** = 120)	Healthy control (***N*** = 60)	***p***-value
	Frequency (%)	Frequency (%)	
**Gender**			
Male	71 (59.2%)	36 (60.0%)	.91
Female	49 (40.8%)	24 (40.0%)	
**Education level**			
Complementary	41 (34.2%)	21 (35.0%)	.73
Secondary	60 (50.0%)	27 (45.0%)	
University	19 (15.8%)	12 (20.0%)	
**Marital Status**			
Single	95 (81.9%)	6 (10.0%)	<.001
Married	9 (7.8%)	52 (86.7%)	
Widowed	2 (1.7%)	0 (0.0%)	
Divorced	10 (8.6%)	2 (3.3%)	
**Monthly income**			
No income	27 (23.3%)	0 (0.0%)	<.001
< 1000 $	61 (52.6%)	39 (67.2%)	
1000–2000 $	26 (22.4%)	12 (20.7%)	
> 2000 $	2 (1.7%)	7 (12.1%)	
**Family history of psychiatric illness**			
Yes	42(36.5%)	5 (8.5%)	<.001
No	73(63.5%)	54 (91.5%)	
	**Mean ± SD**	**Mean ± SD**	
**Age**	48.4 ± 7.6	47.9 ± 7.4	.67
**Duration of illness in years**	20.6 ± 12.4		
**Length of hospitalizations in years**	12.4 ± 8.5		
**Number of hospitalizations**	6.3 ± 5.6		
**Total PANSS scale**	82.8 ± 27.1		
**Positive PANSS**	19.9 ± 9.5		
**Negative PANSS**	17.5 ± 7.9		
**General psychopathology**	45.5 ± 16.8		

### Medications used

The medications of people living with schizophrenia used were typical antipsychotics (76.7%), atypical antipsychotics (50%), mood stabilizers (49.2%), benzodiazepines (37.5%), anticholinergics (70.8%), and antiepileptics (9.2%). Only 5 and 7.5% of the participants were taking SSRI and TCA antidepressants, respectively, and 50.8% were using other types of medications. The mean chlorpromazine equivalent dose was 1041.6 ± 1122.3, and the mean duration of medication treatment was 54.7 ± 29.5 months.

### Comparison of neurocognition and subjective cognitive complaints in patients with schizophrenia and healthy controls

**Fig. 1:**
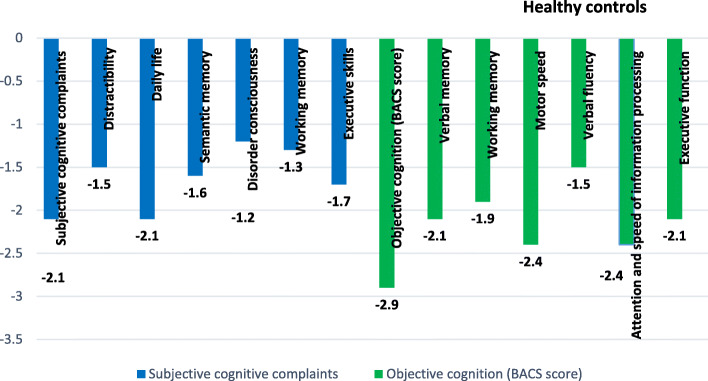
Composite scores for subjective cognitive complaints and objective cognition scores and subtests in people living with schizophrenia compared to healthy controls

The SASCCS total score mean in patients with schizophrenia was 25.15 (SD = 16.67; min = 0, max = 76; median = 23.50). In the healthy control group, the SASCCS total score mean was 9.15 (SD = 7.63; min = 0, max = 37; median = 7.00). A significant difference was found between schizophrenia patients and healthy controls in all neurocognition and SCC tests (*p* < .001 for all) (Table 2). Figure 1 shows the mean composite scores for the SASCCS scale and the BACS total score in patients with schizophrenia compared to healthy controls. A significant difference was found between patients and controls, where the schizophrenia group scored significantly lower mean composite score than the healthy controls in SCC and neurocognition tests in all domains (more deficits) (*p <* .001 for all).

**Table 2:** Difference of objective cognition and subjective cognition between people living with schizophrenia and healthy controls

	People living with schizophrenia (***N*** = 120)	Healthy control (***N*** = 60)	***p-***value
	Mean ± SD	Mean ± SD	
**The Self-Assessment Scale of Cognitive Complaints (SASCCS)**	25.1 ± 16.7	9.1 ± 7.6	< 0.001
Distractibility	4.7 ± 4.4	1.9 ± 1.8	< 0.001
Daily life	4.1 ± 3.5	0.9 ± 1.5	< 0.001
Semantic memory	2.1 ± 2.3	0.5 ± 1.1	< 0.001
Disorder consciousness	3.1 ± 2.7	1.2 ± 1.6	< 0.001
Working memory	4.4 ± 2.8	2.1 ± 1.8	< 0.001
Executive skills	6.2 ± 3.8	2.6 ± 2.2	< 0.001
**BACS score total**	109.9 ± 47.2	221.7 ± 38.5	< 0.001
Verbal memory	20.9 ± 9.6	41.1 ± 9.4	< 0.001
Working memory	10.6 ± 5.5	19.3 ± 4.4	< 0.001
Motor speed	35.7 ± 15.0	70.7 ± 14.4	< 0.001
Verbal fluency	20.4 ± 9.2	34.6 ± 9.2	< 0.001
Attention and speed of information processing	12.2 ± 12.4	38.0 ± 10.7	< 0.001
Executive function	9.9 ± 7.7	17.9 ± 3.7	< 0.001

### Correlations between subjective cognitive complaints and quantitative measures

All objective neurocognition tests were significantly correlated with the total SASCCS scale, except for the attention and speed of information processing. Among the subtests of the SASCCS scale, the working memory was not correlated with the BACS tests.

The total PANSS scale and the general psychopathology PANSS scale were positively correlated with all SCC among patients with schizophrenia. The positive PANSS scale was positively correlated with all subtests of the SASCCS scale, except the disorder consciousness and working memory. The negative PANSS scale was only correlated with the working memory of the SASCCS subtest. The Calgary scale (depression) was positively correlated with the total SASCCS scale and subtests, except the daily life and disorder consciousness SASCCS subscales. The insight scale and the chlorpromazine equivalent dose were not correlated with the total SASCCS scale and subtests (Table 3).

**Table 3:** Pearson correlation between the subjective cognitive complaint (SASCCS), objective cognition (BACS), social cognition, insight, depression, and clinical symptoms in people living with schizophrenia

	SASCCS total score	Distractibility	Daily life	Semantic memory	Disorder consciousness	Working memory	Executive skills
**BACS score total**	−.34***	−.30***	−.36***	−.24***	−.19*	−.07	−.43***
Verbal memory	−.42***	−.35***	−.42***	−.30***	−.26***	−.20*	−.48***
Working memory	−.32***	−.27***	−.29***	−.23*	−.16	−.14	−.41***
Motor speed	−.31***	−.30***	−.34***	−.18	−.23*	−.04	−.36***
Verbal fluency	−.33***	−.31***	−.35***	−.26***	−.13	−.08	−.44***
Attention and speed of information processing	−.07	−.05	−.12	−.06	.02	.05	−.18
Executive function	−.21*	−.18*	−.23*	−.17	−.14	−.03	−.23*
**Total PANSS scale**	.39***	.36***	.33***	.32***	.29***	.23*	.38***
Positive PANSS scale	.23*	.24***	.20*	.18*	.11	.12	.20*
Negative PANSS scale	.18*	.13	.13	.14	.16	.18*	.15
General psychopathology PANSS scale	.42***	.38***	.36***	.34***	.32***	.21*	.42***
**Insight Scale for psychosis**	.08	.06	.12	−.02	.05	.14	.03
Awareness of illness	−.06	−.12	−.02	−.17	.04	.02	−.04
Need for treatment	.04	.06	.05	.04	−.02	.04	.04
Attribution of symptoms	.15	.15	.16	.09	.07	.19	.09
**Depression**	.33***	.41***	.16	.37***	.13	.23*	.23*
**Chlorpromazine equivalent dose**	.12	.05	.18	.06	.13	.05	.17

*p < 0.05; **p < 0.01, ***p < 0.001

The correlation matrix for the scales used in this study is showed in the supplementary Table 5.

Also, higher illness (*r* = .34, *p* < .001) and hospitalization (*r* = .26, *p* = .004) were significantly associated with higher SCC. Age was not associated with SCC (*r* = .09, *p* = .32).

### Bivariate analysis: correlates of subjective cognitive complaint

A higher mean total SASCCS was found in those taking an anticholinergic medication compared to those who do not (M = 27.35 vs. M = 19.80, *p* = 0.010). Gender, education level, and a family history of psychiatric illness did not show any significant association with the SASCCS total scale (*p* > 0.05 for all) (Table 4).

**Table 4:** Bivariate analysis taking the subjective cognitive complaints as the dependent variable in people living with schizophrenia

	SASCCS Total score	
	Mean ± SD	***p-***value
**Gender**		
Male	22.87 ± 16.32	0.072
Female	28.44 ± 16.78	
**Education level**		
Complementary	27.51 ± 18.38	0.172
Secondary	25.51 ± 16.81	
University	18.89 ± 10.27	
**Family history of psychiatric illness**		
Yes	28.71 ± 18.40	0.085
No	23.18 ± 15.54	
**Anticholinergic medication**		
Yes	27.35 ± 17.64	**0.010**
No	19.80 ± 12.72	

Values marked in bold are significant

### Hierarchical regression analysis

A hierarchical regression analysis taking the total SASCCS scale as the dependent variable are displayed in Table 5.

**Table 5:** Hierarchical regression analysis for variables predicting the subjective cognitive complaint (SASCCS total score) among people living with schizophrenia

Variables	Model 1			Model 2			Model 3		
	UB (95% CI)	SB	***p-***value	UB (95% CI)	SB	***p***-value	UB (95% CI)	SB	***p***-value
**BACS**	−.102 (−.169; −.036)	−.289	.003*	−.083 (−.150; −.016)	−.236	.015*	−.065 (−.129; −.002)	−.185	.044*
**PANSS positive**				.109 (−.242;.459)	.062	.540	.023 (−.303;.350)	.013	.887
**PANSS negative**				.125 (−.232;.482)	.060	.489	.074 (−.256;.403)	.035	.659
**PANSS general psychopathology**				.303 (.092;.513)	.305	.005*	.296 (.101;.491)	.298	.003*
**Insight**				1.239 (−.073;2.551)	.158	.064	.762 (−.473;.1.997)	.097	.224
**Depression**							.756 (.271;1.241)	.236	.003*
**Autonomy (total ADL)**							−6.352 (−10.193;-2.512)	−.273	.001*
*R*^2^	.134			.282			.404		
*R*^2^ adjusted	.104			.230			.350		
*F*	4.440*			5.442*			7.397*		
*R*^2^ change	.134			.148			.123		
*F* change	4.440			5.716			11.210		

*UB* Unstandardized Beta, *SB* Standardized Beta, *CI* Confidence Interval

The three models were adjusted for gender, education level and age

*p < .05

In the first step, BACS scores were associated with the SASCCS scale, where a higher BACS score was significantly associated with lower SCC (Beta = −.10, *p* = .003) (Table 5, model 1).

In the second model, when adding the clinical symptoms and insight, the results showed that the BACS remained significant (Beta = −.08, *p* = .015), and higher PANSS general psychopathology (Beta = .30, *p* = .005) was significantly associated with higher SCC (Table 5, model 2). The addition of clinical symptoms and insight showed a significant increase in R2 of 0.148, F change = 5.442, *p* < 0.001 (Model 2).

In the third model, when adding depression and autonomy, the results showed that the BACS total score (Beta = −.06, *p* = .04) and the PANSS general psychopathology (Beta = .29, *p* = .003) remained significant, and higher depression (Beta = .75, p = .003) was significantly associated with higher SCC. However, higher autonomy (Beta = − 6.35, *p* = .001) was significantly associated with lower SCC (Table 5, model 3). The addition of depression and autonomy showed a significant increase in R2 of 0.123, F change = 7.397, *p <* 0.001 (Model 3).

### Structural equation modeling

A Structural Equation Model (SEM) was constructed to demonstrate the regression weight of the relationships between objective cognition, clinical symptoms, depression, insight, autonomy, and subjective cognitive complaints. The results of the model fit indices were as follows: the Maximum Likelihood Chi-Square = 30.58 and Degrees of Freedom = 30, a χ2/df = 1.01. For non-centrality fit indices, the Steiger-Lind RMSEA was 0.132 [0.08–0.18], the GFI, 0.924, AGFI 0.840 and CFI: 0.729.

**Fig. 2:**
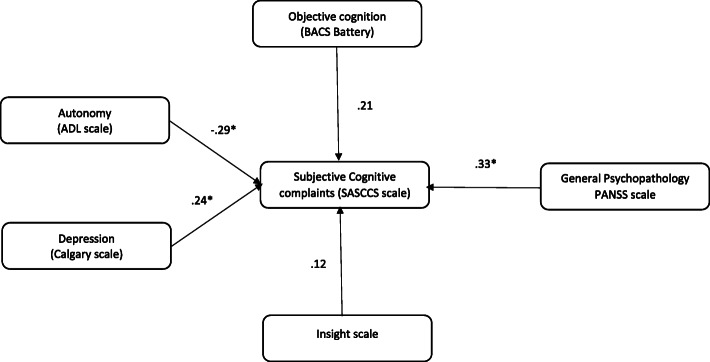
Standardized regression weights of the relationships between neurocognition, clinical symptoms, depression, insight, autonomy, and subjective cognitive complaints. *p < 0.05. Values with * indicate significant associations. BACS: Brief Assessment of Cognition in Schizophrenia, Calgary scale: depression, ADL: activity of daily living, PANSS: Positive and Negative Syndrome Scale, SASCCS: Self-Assessment Scale of Cognitive Complaints in Schizophrenia

The results showed that the path coefficients from depression, objective cognition, autonomy, and general psychopathology to SCC were significant. The two most contributing variables were general psychopathology (Standardized Beta (SB): .33, *p* < 0.001) and autonomy (SB: −.29, *p <* 0.001), followed by depression (SB: 0.24, *p <* 0.001) and objective cognition (SB: − 0.21, *p* = .003) (Fig. 2).

## Discussion

Our study evaluated subjective cognitive complaints (SCC) and their relationship with objective cognitive performance in a sample of in-patients with schizophrenia, compared to healthy controls. It also explored the factors related to SCC among these patients. Self-reporting of cognitive impairment in schizophrenia might be crucial. If patients can reliably describe their own cognitive issues, this should help in the functional outcome and individual treatment interventions. Few studies were available on the treatment of mental disorders in the Arab world, the majority being conducted in community or hospital settings, evaluating psychiatric care, the type of services used and the medical treatment received. Treatment approaches such as social support, psychotherapy, community or government support seem to have a minor role in the management of mental disorders in these countries [41]. Also, there is no specific treatment guide for schizophrenia, the use of psychotropic drugs as part of the treatment of mental disorders is the mainstay of treatment. Moreover, there are a very limited number of psychosocial rehabilitation centers despite an alarming incidence of mental illness in Arab countries. Lebanon has neither a mental health policy nor a truly active mental health program. The mental health problem in Lebanon has long been recognized and there is a need for planning and monitoring of services and evaluation of the quality of mental health services [42].

The results of our studies have shown that people living with schizophrenia complain about their cognition in the broad sense and can estimate their cognitive impairment, regardless of their level of insight in agreement with previous findings [4, 7, 15, 43, 44]. The explanation could be that patients with schizophrenia might experience and report difficulties with their cognitive processing on one side, and on the other side, healthy controls are aware they do not exhibit deficits in their cognitive performance. However, when the SASCCS total scale and subscales were correlated with corresponding neuropsychological tests in patients with schizophrenia, a negative association was found between all the measures, except for those relating to attention and speed of information processing. Stip et al. had found similar results among 114 patients with schizophrenia, showing negative correlations between subjective scores and objective cognitive assessment in several domains [7]. In a more recent study among 100 people living with schizophrenia, Baliga et al. showed that of all cognitive domains, only working memory test scores correlated positively with SCC [15]. Other studies revealed conflicting results, where objective and subjective cognition scores did not correlate [4, 11, 45, 46]. A possible explanation of our findings could be that the more the patients have an objective cognitive deficit, the more they complain about it, indicating that they might be aware of their deficit and could subjectively express their cognitive functioning. Another interpretation could be that patients whose abilities are unimpaired might overestimate their problems. Additionally, variations of results across studies could be due to the different tests used to assess the objective and subjective cognitive performance.

The results of this study showed that SCC was related to clinical symptoms essentially the general psychopathology subscale. Consistently, Lecardeur et al. have found that among 176 patients with schizophrenia a positive association between the PANSS cognitive factor and the SCC [43]. In contrast, a study conducted in Tunisia among 105 patients with schizophrenia found no correlation between the SASCCS score and the PANSS cognitive factor [29]. Also, Baliga et al. could not find a relation between the severity of symptoms and SCC; nevertheless, SCC correlated with individual items of the PANSS scale [15]. In our study, patients who had more psychotic symptoms were more likely to complain about their cognition. In addition, negative PANSS subscale was weakly and positively correlated with subjective cognitive complaint. In line, a study done by Harvey et al. among 177 people living with schizophrenia have found that the autistic traits measured by the negative items of the PANSS were associated with an overestimation of social functioning and social cognitive ability. People living with schizophrenia might have an impairment of reporting their social cognitive abilities, they have either an overestimation or underestimation of their own performance [47]. These patients have problems in subjective cognitive assessments and self-evaluation of their social cognitive abilities and everyday social consequences [47]. Psychotic patients and those at risk of exhibiting psychotic symptoms showed to be less confident in their cognitive abilities [48].

Our study could not find a correlation between SCC and insight, confirming previous results showing that patients with schizophrenia might be aware of their cognitive deficits despite having no insight into their condition or symptoms [15, 49–51]. Patients with schizophrenia still do not feel the need for treatment for their psychotic symptoms; likely, they do not see the need for help for their cognitive function due to a lack of insight [1]. Therefore, the consciousness of cognitive deficits might occur without an understanding of the disease symptoms, independent of insight. This supports the idea that insight is a multi-dimensional phenomenon with biological mechanisms that are likely to be independent of one another [52]. Our study also suggests that insight has less predictive value for SCC once the depression variable is included in the regression model, similar to the findings of Sellwood et al. in a study among 115 patients with schizophrenia [4]. Our results showed a positive association between the SASCCS total score and the Calgary scale, indicating that the more the patients have depressive symptomatology, the more they report cognitive difficulties, consistent with the Tunisian study that found a positive correlation between the SCC and depression as measured by the Calgary scale [29]. Other studies also demonstrated that among PANSS items, higher SCC correlated with higher depression [15, 43]. A possible explanation for this correlation is that individuals with depression may be excessively sensitive and are more likely to attribute their symptoms on impaired cognitive disorders, in line with a large body of evidence showing that depression is linked to a desire to attribute negative experiences to internal causes [53].

Moreover, our results showed that low autonomy is associated with high cognitive complaints. Few studies have examined the relationship between activities of daily living and subjective cognitive complaints in schizophrenia. Baliga et al. found a negative correlation between SCC and social functioning, particularly occupational and other social roles, among 100 patients with schizophrenia [15]. Other studies among elderly patients showed an association between higher SCC and lower daily living activities [54–56]. Functional decline, such as sedentary behavior with low physical activity, is common among patients with schizophrenia [57]. Among this group, a vicious cycle of physical dysfunction and low activity levels, exacerbated by symptomatic and cognitive deficits, can result in significant daily functioning impairment [57]. The association between changes in daily activities and cognitive functions in schizophrenia patients remains unknown; thus, more studies are needed to elucidate this association.

Our findings revealed that the two most contributing variables of SCC were general psychopathology and autonomy, followed by depression and objective cognition. No similar framework has been found in the literature that explores the relationships between these factors and SCC. A possible interpretation of our results could be that the general psychopathology subscale of the PANSS consists of several cognitive deficits, such as disorientation, poor attention, lack of insight, and active social avoidance [33], explaining the overlapping dimensions with the SCC. Additionally, patients who experience problems in their daily activities may have a frequent complaint about their cognition. Further studies are needed to clarify the interactions between the identified variables and the SCC.

### Implications of the current study

Cognitive remediation therapy is a behavioral training–based intervention for schizophrenia that seeks to enhance cognitive functions with the objective of long-term maintenance and generalization [58]. Four key aspects for cognitive remediation were recognized in a recent expert consensus: the presence of an engaged and qualified therapist, frequent practice of cognitive exercises, organized development of cognitive strategies, and the application of methods to promote the transfer of cognitive gains to the real world [58]. A recent systematic review and meta-analysis including 130 studies have found that the four core elements of cognitive remediation significantly produced larger benefit on cognitive and functional outcome [59]. However, treatment duration was directly associated only with functional gain but not with cognitive outcome [59]. In addition, atypical antipsychotic treatments have been shown to improve objective cognitive impairments and this impact may also translate to subjective cognitive impairments, according to some evidence [60]. Studies have showed that cognitive impairment perceived by schizophrenia patients has been linked to objective neurocognitive tests.

Studies carried out on subjective complaints have the interest of evaluating the capacity of patients to know their cognitive disorder and can help them in the implementation of specific cognitive remediation activities but also training in social skills and development. As a result, these assessments help determine the altered and preserved skills for each patient. The results of the assessment are linked to the patient’s functioning before any referral to a rehabilitation program, the main purpose of which is to reduce the effects of the disease. For each patient, precise reintegration goals should be determined based on the results of the cognitive assessment. In addition, in patients with a subjective cognitive complaint, it seems interesting to assess the autonomy to set up specific cognitive remediation programs that help to train them on their social skills and improve their autonomy.

However, psychiatric hospitals in Lebanon do little work on treatments for cognitive deficits in schizophrenics, so it would be interesting to encourage clinicians in hospitals to start studying the cognitive aspect in schizophrenics to reduce the impact of the disease and allow improved care and thus deinstitutionalization of patients suffering from schizophrenia. The interest therefore in the study of cognitive functions is to lead to a rehabilitation that aims to allow schizophrenic patients to reintegrate into society.

### Limitation

This study has several limitations. First, it is not possible to analyze and understand a complaint when it is hidden by a lack of insight or when the instrument used to measure insight is not appropriate. Second, the cross-sectional design makes it difficult to find causal relations between SCC and related factors. Third, the results could not be generalized to the population because of the small sample size, with patients selected from one site. In addition, the healthy control group was not exactly matched to the patient group, it was likewise small (*n* = 60). Also, the population consists of chronically hospitalized patients whose cognitive function might be severely impaired, which may lead to a selection bias. Information bias is possible as the information was self-reported by the participants; accurate details could not be provided during the face-to-face interview. Residual confounding bias could have occurred since not all factors related to SCC were tested.

## Conclusion

In conclusion, our results suggest that a significant proportion of patients with schizophrenia can estimate their cognitive impairment, independent from their level of insight, as a correlation was found between the objective cognitive measures and the subjective cognitive test. Also, a positive correlation was found between depression and SCC, suggesting that this aspect should be investigated along with the clinical symptoms when a patient with schizophrenia presents with SCC. The relations between activities of daily living and SCC require further investigation to identify the risks of further decline and provide the needed support for people living with schizophrenia experiencing ADL difficulty. Subjective cognitive complaints are becoming more widely recognized as indicators of potential cognitive dysfunction, with evidence suggesting such complaints are a precursor to cognitive impairment. In addition to depression or objective cognition, it seems interesting to assess autonomy in patients with SCC to set up specific cognitive remediation programs that help training their social skills and improve their autonomy. The subjective assessment of cognitive functions may give a more comprehensive view of an individual’s cognitive profile; however, it cannot replace the objective assessment measures as the self-report of neurocognitive functioning might not be accurate. Also, it will be of interest to develop pharmacological and psychological therapies to help this population improve their cognitive performance.

Further prospective studies with a larger sample size are necessary to fully understand the SCC among patients with schizophrenia to help remediate impairments in everyday functioning.

## Supplementary Information

**Additional file 1.**

## Data Availability

The datasets used and/or analysed during the current study are available from the corresponding author on reasonable request.

## Abbreviations

SCC: Subjective cognitive complaints
SSTICS: Subjective Scale To Investigate Cognition in Schizophrenia
SASCCS: Self-Assessment Scale of Cognitive Complaints in Schizophrenia
HPC: Psychiatric Hospital of the Cross
DSM: Diagnostic and Statistical Manual of Mental Disorders
BACS: Brief Assessment of Cognition in Schizophrenia
PANSS: The Positive and Negative Syndrome Scale
CDSS: Calgary Depression Scale for Schizophrenia
ADL: Activities of Daily Living
IS: Insight Scale for Psychosis
SEM: Structural equational model
RMSEA: Root Mean Square Error of Approximation
GFI: Goodness-of-fit statistic
AGFI: Adjusted goodness-of-fit statistic
CFI: Comparative fit index
