# Supplementary material for: Subjective cognitive complaints and relations to objective cognitive performance among Lebanese patients with schizophrenia
Source: BMC Psychiatry. 2021 Nov 9;21:549. doi: 10.1186/s12888-021-03564-w (PMC8576858; doi:10.1186/s12888-021-03564-w)
Supplement: Supplementary file 1 — Additional file 1. [file 12888_2021_3564_MOESM1_ESM.docx]

**Supplementary file**

| **Supplementary table 1**  **Factor analysis of the Self-Assessment Scale of Cognitive Complaints in Schizophrenia (SASCCS) among people living with schizophrenia** | | | | | | | | | |
| --- | --- | --- | --- | --- | --- | --- | --- | --- | --- |
|  | **Items** | **Factor 1** | **Factor 2** | | **Factor 3** | | **Factor 4** | | **Factor 5** |
| Do you have difficulties to organize your daily activities? Such as shopping, cooking, cleaning the house, fixing stuff, doing some laundry | 18 | 1.018 |  | |  | |  | |  |
| Do you have difficulties planning something in advance? Example, updating your health care card, getting some money from your post office account, or planning how to spend your budget for the month? | 17 | .818 |  | |  | |  | |  |
| Do you have difficulties to do usual activities? Example, to dress up or button a shirt, to introduce a key in a lock, to use a spoon? | 21 | .699 |  | |  | |  | |  |
| Do you have difficulties to focus on something for more then 20 minutes? Example, listening to the news, reading a magazine, watching a sitcom, attending a school lesson. | 16 | .620 |  | |  | |  | |  |
| Do you have difficulties to change your way of thinking or your manner of doing something the way you’re used to do it when you’re asked to do so and you agree to make these changes? | 19 | .546 |  | |  | |  | |  |
| Do you feel like you have memory disturbances? | 1 |  | .942 | |  | |  | |  |
| Do you have difficulties to retain something in your mind? Example, a shopping list or a list of persons’ names | 3 |  | .894 | |  | |  | |  |
| Do you have any problems to remember information you’ve just learned and that you should immediately use? Example, an address, a telephone number, a bus number, a doctor’s name | 2 |  | .887 | |  | |  | |  |
| Do you have any problems to remember information you learned in a paper or watched on TV yesterday? | 7 |  | .466 | |  | |  | |  |
| Have you ever forgotten how to cook a dish or which ingredients you should put in / Have you ever forgotten how to fix or repair things at home | 8 |  | .419 | |  | |  | |  |
| Do you have any problems remembering names of people belonging to fields you’re usually interested in? (sports, cinema, songs…) | 10 |  | .400 | |  | |  | |  |
| Do you have any difficulties staying in alert and reacting quickly when something you didn’t expect happens? Example, avoiding a car when crossing the street | 13 |  |  | | .875 | |  | |  |
| Do you have any problems remembering names of the biggest towns in Tunisia or the most important historical events of your country, or the names of the biggest cities in the world? | 11 |  |  | | .737 | |  | |  |
| Do you have difficulties to find your words, to make sentences, to understand the meaning of some words, to pronounce them, to designate objects by their name | 20 |  |  | | .694 | |  | |  |
| Do you feel like you are distracted for example when speaking with someone or reading a magazine? | 12 |  |  | | .687 | |  | |  |
| Have you ever forgotten an appointment with your friend or with your doctor? | 5 |  |  | | .606 | |  | |  |
| Do you have any problems to find your way by yourself to the hospital, the outpatient clinic or even to your home? | 9 |  |  | |  | | .854 | |  |
| Do you have difficulties to do 2 different things at the same time? Example, having a conversation with someone while watching television, or doing some housekeeping while cooking a lunch on the gas stove | 15 |  |  | |  | | .835 | |  |
| When the television is on and people around are talking loudly, do you have any difficulties to focus on a particular conversation? | 14 |  |  | |  | |  | | .877 |
| Do you sometimes forget to take your treatments? | 6 |  |  | |  | |  | | .441 |
| **Cronbach’s alpha** |  | .91 |  | |  | |  | |  |
| **Percentage of variances explained** |  | 65.19% |  | |  | |  | |  |
| **Supplementary table 2**  **Factor analysis of the Calgary Depression Scale for Schizophrenia (CDSS) among people living with schizophrenia** | | | | | | | | | |
|  | | | | **Items** | | **Factor 1** | | **Factor 2** | |
| MORNING DEPRESSION: When you have felt depressed over the last 2 weeks have you noticed the depression being worse at any particular time of day? | | | | 6 | | .834 | |  | |
| DEPRESSION: How would you describe your mood over the last two weeks? Do you keep reasonably cheerful or have you been very depressed or low spirited recently? In the last two weeks how often have you (own words) every day? All day? | | | | 1 | | .791 | |  | |
| SELF DEPRECIATION: What is your opinion of your self compared to other people? Do you feel better, not as good, or about the same as others? Do you feel inferior or even worthless? | | | | 3 | | .681 | |  | |
| EARLY WAKENING: Do you wake earlier in the morning than is normal for you? How many times a week does this happen? | | | | 7 | | .669 | |  | |
| HOPELESSNESS: How do you see the future for yourself? Can you see any future? - or has life seemed quite hopeless? Have you given up or does there still seem some reason for trying? | | | | 2 | | .591 | |  | |
| GUILTY IDEAS OF REFERENCE: Do you have the feeling that you are being blamed for something or even wrongly accused? What about? (Do not include justifiable blame or accusation. Exclude delusions of guilt.) | | | | 4 | |  | | .983 | |
| PATHOLOGICAL GUILT: Do you tend to blame yourself for little things you may have done in the past? Do you think that you deserve to be so concerned about this? | | | | 5 | |  | | .701 | |
| SUICIDE: Have you felt that life wasn’t worth living? Did you ever feel like ending it all? What did you think you might do? Did you actually try? | | | | 8 | |  | | .594 | |
| OBSERVED DEPRESSION: Based on interviewer’s observations during the entire interview. The question “Do you feel like crying?” used at appropriate points in the interview, may elicit information useful to this observation. | | | | 9 | |  | | .423 | |
| **Cronbach’s alpha** | | | | | | .83 | |  | |
| **Percentage of variances explained** | | | | | | 56.48% | |  | |

| **Supplementary table 3**  **Factor analysis of the Insight scale among people living with schizophrenia** | | | |
| --- | --- | --- | --- |
|  | **Items** | **Factor 1** | **Factor 2** |
| I do not need medication | 3 | .834 |  |
| I do not need to be seen by a doctor or psychiatrist. | 6 | .774 |  |
| The doctor is right in prescribing medication for me | 5 | .749 |  |
| My stay in hospital is necessary | 4 | .670 |  |
| Some of my symptoms are made by my mind. | 1 | .247 |  |
| None of the unusual things I experience are due to an illness | 8 |  | -.715 |
| I am mentally well. | 7 |  | .628 |
| If somebody said that I have a nervous or a mental illness then they would be right. | 2 |  | .617 |
| **Cronbach’s alpha** | | .50 |  |
| **Percentage of variances explained** | | 46.69% |  |

| **Supplementary table 4**  **Factor analysis of the Activities of Daily Living (ADL) scale among people living with schizophrenia** | | | |
| --- | --- | --- | --- |
|  | **Items** | **Factor 1** | **Factor 2** |
| Dressing | 2 | .815 |  |
| Personal hygiene | 1 | .780 |  |
| Functional mobility | 4 | .774 |  |
| Toilet hygiene | 3 | .753 |  |
| CONTINENCE | 5 | .612 |  |
| Self-feeding | 6 |  | .965 |
| **Cronbach’s alpha** | | .68 |  |
| **Percentage of variances explained** | | 64.71% |  |

| **Supplementary table 5**  **Correlation between the scales used in this study among people living with schizophrenia** | | | | | | |
| --- | --- | --- | --- | --- | --- | --- |
|  | **SASCCS total score** | **BACS score total** | **Total PANSS scale** | **Insight Scale for psychosis** | **Depression** | **Autonomy** |
| **SASCCS total score** | - | -.34*** | .39*** | .08 | .33*** | -.39*** |
| **BACS score total** | -.34*** | - | -.14 | .19* | -.02 | .24** |
| **Total PANSS scale** | .39*** | -.14 | - | -.02 | .16 | -.10 |
| **Insight Scale for psychosis** | .08 | .19* | -.02 | - | .01 | -.13 |
| **Depression** | .33*** | -.02 | .16 | .01 | - | -.13 |
| **Autonomy** | -.39*** | .24** | -.10 | -.13 | -.13 | - |
| *p<.05 ; **p<.01, ***p<.001 | | | | | | |
